# Bibliographical Notices

**Published:** 1840

**Authors:** 


### Periscope;

OR,

### CIRCUMSPECTIVE REVIEW.

" Ore trahit quodcunque potest, atque addit acervo."

### Notices of some New Works.

Parochial Medical Relief, considered in a Letter to the "Poor-Law Commissioners," developing an entirely New System of Medical Remuneration, alike conducive to the Interests of the Rate-payers, the well-being of the Poor, and the respectability of the Profession. By *E. T. Meredith*, Member of the Royal College of Surgeons, London. Pp. 36. Highley, 1840.

The question of Parochial Medical Relief has certainly lost nothing of its importance, and would appear to have lost little of its complexity. After all the evidence that has been taken, and the experience that has been obtained, we have before us what we believe to be a novel proposition, candidly urged and ingeniously supported by a gentleman who appears to be practically well acquainted with the subject.

When the Poor-Law Commissioners began their unjust and tyrannical crusade against the medical profession, we told them that it was no less impolitic than wrong, and would probably be as unsuccessful as impolitic. As if it were not enough to inflame the prejudices and outrage the feelings of the lower classes, they must array against the law which they misapplied, the influence of an insulted and a powerful profession. The Commissioners were warned of this, but they persisted in their contumelious course, and much of the difficulty which the Poor Law has encountered may be traced to their perverse, we might almost say their insolent, obstinacy.

No honourable man in the profession contends that medical relief to the poor should be looked on essentially as a medical question. It is in all its length and breadth the poor man's question. It affects him in the most vital way, for on its right solution hang his health and strength and life. If those on whose skill his fate depends are so insufficiently remunerated, that their interests lead them to neglect him, he knows enough to be well aware that neglected he *must* be. The motives that influence bodies of men are no secret, human nature is not altered by the lawyer's coif or the doctor's degree, and though individual instances of extraordinary, or, perhaps, quixotic benevolence, may stand out here and there beyond the common level of the feelings of the community, these must not be allowed to form the elements of selfish calculations.

The abominations of the "Tender System" have, we trust, passed away—the Commissioners, we hope, have ceased to protest that the minimum of medical remuneration is the maximum of perfection of the poor-law—well paid themselves for *their* philanthropy, they have been forced to the acknowledgment that the medical profession is to be no loser by its labours—and the problem which now waits solution is, how the public interest may fairly be consulted with a due regard to that of the poor and the profession. Like all questions of detail this does not admit of a speedy, scarcely of a satisfactory settlement.

Without going into the various plans that have been latterly proposed, to most of which, indeed, we recently devoted some attention, we think it only right to bring Mr. Meredith's under the notice of our readers. As we cannot pretend to a nice or a practical acquaintance with this important matter, we shall content ourselves with bringing forward the main features of Mr. Meredith's proposals, and enabling our readers to form their own judgment upon them.

He entertains some strong objections to the system of paying by the *case*, and by *family tickets*. This plan, if persevered in, will, he is convinced, lay open a wide field for the exercise of intrigue, and prove a fertile source, nay, offer a premium upon the accomplishment, of fraud; for the object of the ill-paid medical officer will assuredly be to increase the number of applicants, and so *impose* on the rates; and the object of the relieving officer will be to protect the rates, and so *deprive* the sick poor of that relief to which the law entitles them. This conclusion may be questioned—it may be said that the character of the profession is above such a suspicion—that the relieving officer can have no motive for so abusing his trust;—nevertheless it must be admitted that the system is open to this most serious objection. How desirable then any plan must be that tends to relieve the profession from the imputation of selfish, mercenary, and faithless conduct—and the relieving officer from such an embarrassing position—and also removes the source of that interminable contention inseparable from such contingencies, and which cannot be otherwise than prejudicial to all parties interested in the contract.

Prior to developing his scheme of medical remuneration, Mr. Meredith is at the pains to define the duties of medical officers.

"The duties of medical officers to Unions, (trusses and unusual bandages being paid for at cost price,) should be to attend upon, and furnish all necessary medicines and surgical assistance, (including operations* and vaccination), to the sick *paupers*, disabled by accident or otherwise, residing in the Union, *upon their own application;* each officer certifying every case as it occurs, in his own district, to the relieving officer, who should as early as possible, visit the same, in order to detect any imposition that may be practised on the part of the applicant, and check any unnecessary attendance on the part of the medical officer; —an exception must be made of difficult midwifery cases, for which an *extra* fee of 10s. 6d. should be allowed;—other cases of sickness, or accident, happening to those not strictly paupers, and not being in the receipt of more than 14s. per week, but who have an order for medical relief from the relieving officer, or any authorised person, guardian, or overseer, should be equally attended to; these cases not being charged without producing the authorised orders. Others again whose income does not exceed 1*l*. 1*s*. a week, should be entitled to the same attendance, &c., by obtaining a loan ticket, for which they should pay 7*s*. 6*d*.; (if midwifery 10*s*. 6*d*. by giving one month's notice, and paying 5*s*. in advance; or 1*l*. 1s. on the emergency); to the relieving officer, to be paid by him to the medical officer, who will return the ticket as a receipt; these loan cases, of course, will not become chargeable to the Union, further than being considered as forming part of the annual average number of cases. In order to prevent any equivocation, as to the *cure* of a case, and repeated attacks of the same, or the accession of another disease, in the same person; I

* "I have seen no good reason alleged, why an extra fee should be allowed for operations—such however has been suggested by many, for whose opinions I entertain a high respect; notwithstanding which I cannot assent to their views on this occasion."

propose that a *case* should be considered as lasting three months, after the expiration of which, the application must be renewed, if the case continues, or a fresh illness arises, but the same individual should not be charged more than twice in the year. The medical officer should as now make a weekly return of the sick under his treatment, accompanied with such observations as the cases call for; which return should be examined by the Board, and certified by the Chairman as approved, or otherwise, as the case may be; but all other reports, involving opinions not *immediately* connected with cases under his care, he should not be expected to make, without receiving a proportionate fee for his trouble. Some provision should be made to ensure *promptitude*, in the execution of the necessary orders for extra diet, &c., issued by the medical officer;—this at present is badly arranged—would it not be well to allow the medical officer to send his orders direct to the parties contracting for the several articles, at least in those cases, where the applicants are receiving pauper relief? The medical districts should be limited to a surface of forty square miles; and no district should furnish on an average more than 600 cases, nor less than 50, annually; neither should a district of forty square miles exceed 300 cases annually—thirty miles 400, and other districts in the same proportion.* I have made no separate provision for attendance, &c., at the workhouse, believing the duties thereof may safely be included in the general arrangement."

The *Remuneration* of those officers is set forth in the following Propositions, which we find it utterly impossible to abridge.

"First,—That the Medical Officer should be remunerated by a 'fixed annual salary,' and a 'sum,'—proportioned to the number of cases attended during the year;—but, as the amount of this 'sum' cannot be ascertained before the end of the year, and as the payments are made quarterly, I would advise a portion, from three to five pounds less than the average, to be paid with the quarterly 'fixed salary,' leaving the balance to be adjusted at the last quarter when the total number of cases will be known.

Second,—That the 'fixed salary' should consist of two sums;—the first, depending upon the *extent* of the *district*, and the progressive *value* of the whole of the *cases* occurring therein, and which may be considered as 'milage;' the second depending upon the annual *number* of cases, (the *value* and *number* being taken on an average of the three preceding years)† and which may be regarded as a 'per centage' on stock in trade.

The 'fixed salary' thus established will guarantee the medical officer from loss; (it is much to be feared that Boards of Guardians attach little import to this), the necessity of which will perhaps more readily be conceded, when I affirm that the expenses‡ incurred in conducting a country private practice of 300 cases

---

* "It has been proposed to found the calculation of the value of medical salaries, upon the extent of the district, and the amount of population; this principle, however, can never safely be applied, because the sickly, or healthful character of a district may be quite uninfluenced by the amount of its population; again, the population may be comparatively rich or poor.—An area of forty square miles I know is by many considered too extensive, but with the reservations here laid down I think the objection vanishes."

† "In adopting this system where the remuneration has been by a sole annual salary, the first year will necessarily be experimental in regard to the average, as the present returns will scarcely afford a just criterion of the number of bona-fide cases; taking this into consideration, I therefore think that ONE FOURTH of the reported cases may be safely and fairly deducted from the three preceding annual returns and the average taken on the remaining three fourths."

‡ These expenses do not include the CAPITAL sunk in education, &c. the interest on which cannot be estimated at less than £50. per annum."

annually, averaging 12s. 6d. per case; and still more apparent will the necessity be to guard against anything like risk, when it cannot be disguised, that as surely as the medical officer finds the chances running against him, so certainly will the poor be neglected.

Third,—That the 'milage' should be determined at a rate per square mile, *progressively decreasing* as the miles *increase*. That this *rate* should be such as to give for forty square miles of surface yielding 200 *cases* annually, the sum of £40.

A progressive rate is absolutely necessary, to afford a fair estimate of the value of the *outlay* and *labour*, required to work unequal surfaces; and the same is equally applicable in respect to unequal numbers of cases. I have assumed, that to work forty square miles of surface yielding 200 cases annually, it will be worth £40. Granting this,—which is but reasonable,—I find by arithmetical progression, that the value of the first mile descending, will be £1. 19s. 0¼d. $\frac{7}{41}$f. and that the last will be only of the value of 11½d. $\frac{34}{41}$f. I shall perhaps better explain myself by the following example. If forty miles be worth £40. to work, twenty miles will be worth £29. 15s. 1¼d. by progression, instead of £20, by simple proportion;—in the same ratio, ten miles will be worth £17. 6s. 4d.—and five miles £9. 5s. 4d. By the same rule the value of any given surface may be found.

Should this proportion be objected to, as not adapted to all circumstances, an uniform rate of £1. 5s. per square mile would best meet the difficulty, and would perhaps give more general satisfaction; at the same time I am rather inclined to adhere strictly to the progressive rate, as being far more equitable. For although certain expenses may be incidental to the performance of a given work, over a given surface, it does not follow that those expenses will preserve an equal or simple proportion to any other space, having the same duties, much less if those duties are not uniform. One horse may be equal to forty miles; but one horse must be had to do twenty;—therefore for working twenty miles, you ought to receive more than one half the value of forty miles, the *expenses* being nearly the same, notwithstanding, that the *labour* may be diminished exactly one half. I have said the 'milage' should have reference likewise, to the progressive value of the whole of the cases, (forming the 'fluctuating salary' which will be described hereafter), and this rather than simply to the number; inasmuch as each individual case will be of unequal value. To illustrate this, I assume 200 cases occurring in a district of forty square miles, for attendance on which, *in that space*, I allow £40. as 'milage,'—but as 200 cases will give £40. for forty miles, 300 cases would give £60. for the same space by simple proportion;—this, however, is too much, because the additional 100 cases, would not increase the *expenses* or *labour*, by one half; i. e. in the same proportion. I therefore say, let the 'milage' previously determined for any given surface, bear also a simple proportion to the *progressive value* of the whole number of cases; 200 being the standard for any district. I fix upon 200 because that number will average nearly 12s. per case* on the whole annual receipt in a district of forty square miles: which I consider as only a fair remuneration for the faithful attendance on, and the due supply of medicine (during one year) for, 200 *bona-fide* cases of sickness, or accident happening in a district of that extent; e. g. as £49. 19s. 4d. the progressive value of 200 cases, is to £67. 9s. 3d. the value of 300 cases; so will £40. the value of the milage for forty miles, with 200 cases, be to £54. 0s. 1¼d. the value of the milage sought for forty miles, with 300 cases;—

(£49. 19s. 4d. : £67. 9s. 3d. : : £40. : £54. 0s. 1¼d.).

* "It will rarely happen that the average per case will exceed 12s. 6d. if this however should appear to be PERMANENT, the district may be altered to meet the difficulty, or the 'per centage' may be reduced."

instead of £60, which simple proportion would give: the milage then for forty miles, averaging 300 cases annually, will be £54. 0s. $1\frac{1}{4}$d.; for the same space with 100 it will be £21. 19s. $11\frac{1}{2}$d.

Fourth,—That the amount of the second portion of 'fixed salary,'—the 'per centage,'—should be estimated at the rate of £15. per cent. on the average annual *number* of cases.

This portion of the 'fixed salary' does not require a progressive rate, as the 'stock' in trade necessary to be kept, will nearly always bear a direct proportion to the number of cases. A valuable feature in the operation of this portion of 'fixed salary,' will be found in its reducing the *difference* of value between each case in the lowest degree, consistently with the value (£180.) of the whole 600 cases herein assumed; and yet the *difference* will be great enough to preserve the principle of the progressive rate,—e. g. the first of 600 cases according to this *joint system,* will be worth 8s. $11\frac{3}{4}$d. $\frac{313}{601}$f. and the last will be worth 3s. 0d. $\frac{288}{601}$f.;—according to *progression only,* the first of 600 cases will be worth 11s. $11\frac{3}{4}$d. $\frac{25}{601}$f., the last being of the value of $\frac{576}{601}$f. This will appear more striking if we calculate upon 200 cases; the value of the first being, by my system, (avoiding fractions) 9s. and the last 7s. averaging 8s. per case;—by progression *only* the value of the first of 200 cases will be 12s. and the last 8s. averaging 10s. per case;—the difference between the first and the last case, in the first instance being 2s. and in the last 4s.

Should the estimates I have formed be considered too high, the per centage here advised may be reduced, but the integrity of the other financial parts must be preserved, at least so far as not to *reduce* the data on which they are founded.

Fifth,—That the remaining 'sum,'—which may very appropriately be called the 'fluctuating salary,'—should be ascertained by the same rule as was applied to the 'milage,'—i. e. the *value* of each case progressively decreasing as the number of cases increase; the amount of this 'sum together,' with that of the 'fixed salary,' jointly constituting the aggregate annual remuneration.

I have limited the number of cases which any district ought to supply, to the extent of that district: I likewise limit the number of cases which any one medical officer should have under his charge to 600 annually. These limitations I think most essential to the perfect working of this system, and therefore it is most important that they should be enforced as nearly as possible; this arrangement should not however preclude a firm from holding two districts; neither should it debar the same medical officer from the same privilege, provided the *two districts jointly,* do not transgress the limitations above set forth.

Supposing the 600 cases to occur in a town district, where the milage would be little or nothing;* I think £180. would be a fair compensation for attendance, &c. thereon, for one year, being at the rate of 6s. per case; this being a little more than the average expenses of dispensary practice; and less than the value of each case as estimated by the medical witnesses, in their evidence before the select Committee of the House of Commons.† The 'per centage' to conduct these 600 cases will amount to £90, this forming the 'fixed salary,'—leaving £90. (the progressive value of the cases), as the 'fluctuating salary.' Now, if 600 cases produce £90. each case progressively decreasing in value; the first case will be worth 5s. $11\frac{3}{4}$d. $\frac{313}{601}$f. and the last $\frac{288}{691}$f.; the 'aggregate salary' then for 600 cases will stand thus:—

---

* "'Milage' need not be allowed in districts whose area does not exceed two square miles."

† "See likewise the Petition of the medical practitioners in Gloucester, on the subject of payment per case. In reference to this valuable petition, I here take the opportunity of expressing my concurrence in its general prayer."

| | | | | | | |
|---|---|---|---|---|---|---|
| Fixed Salary.......... | £90. | } Aggregate Salary........ | £180. | | | |
| Fluctuating Salary .... | £90. | | | | | |

For 400 it will be :—

| | £ | s. | d. | | £ | s. | d. |
|---|---|---|---|---|---|---|---|
| Fixed Salary...... | 60 | 0 | 0 | } Aggregate Salary | 139 | 19 | 4." |
| Fluctuating Salary | 79 | 19 | 4 | | | | |

Such is the scheme of Mr. Meredith, and we leave it in the hands of the professional public. It may be rather difficult to put in practice, but certainly it seems exceedingly fair to all parties.

---

Illustrations of the Comparative Anatomy of the Nervous System. By *Joseph Swan*. Part V. Price 7s.

This admirable dissector and excellent comparative anatomist continues the work before us. It will form a valuable addition to our stock of facts in the anatomy of the nervous system, and must redound to the permanent reputation of its author. We regret the delay that has occurred in this brief notice of the present part. Better late than never.

There are two Plates (the 26th and 27th). The first exhibits the sympathetic nerve in the right side of the calf—the cervical part of the sympathetic of the fox—the abdominal plexus of the dog.

Mr. Swan makes some observations on the connexion between the thinking principle, whatever that may be, and the material organ, which we have much pleasure in noticing. It goes to disprove the popular and most injurious notion, that anatomical pursuits dispose to materialism in its gross and repulsive form.

"It has been stated," says, Mr. Swan, "that the basis of the nervous system is the nervous element modified by the different substances of the brain, the spinal marrow, and the ganglions; it must, however, approach to the nature of the immaterial being, as it forms the link which unites this with the material organs and through these with the material world, every part being duly sustained by the vital influence of the blood. But some portions of the nervous system minister to its power in a much greater degree than others, and particularly the convolutions of the brain, these are proportionate with the intellectual faculties, to which they give them a wider or more limited range, according to their development; they do not, however, form any part of the immaterial being, but are the mere instruments for its use and power, and necessary for its improvement and enlargement, for it remains mute and passive, exalted or debased, according to its neglect or cultivation, or improper use through these means. Besides the special influence admitted from the senses, it can, for particular purposes, through the material organs, call into action various parts of the body, and render these subservient to its power, in a manner unattainable by similar ones in animals, which are incapable of being raised above their natural state, except in a very slight degree."

"The brain," he goes on to say, his language perhaps being open to some objections, "is the medium through which the mechanical forms, admitted by the eye, are conducted, as well as the different forms of the air in sounds by the ear, and the influence of the organs of the other senses; but the power of comparing and arranging these impressions in thinking, is independent of the material organs, and is an operation of the mind. But although the mind may seem to have been produced, it has only been instructed, and had its powers made apparent through the healthy brain, receiving impressions from without, and imparting others by the nerves to various organs of the body, and producing speech, writing and other accomplishments."

"The mind may seem to be annihilated by apoplexy, or to be perverted in madness, but these are only defects of manifestation, arising from a derangement of the parts of the brain forming the medium of communication between the immaterial being and the material world; it is possible, however, that the mind may be primarily disordered, and involve secondarily the material organs.

After the extinction of the nervous element at death, the immaterial being, or that termed animus by Cicero, still lives, but ceases to will or receive impressions through the material organs. 'Mihi quidem nunquam persuaderi potuit, animos, dum in corporibus essent mortalibus, vivere; cùm exissent ex iis emori: nec verò tum animum esse insipientem, cùm ex insipienti corpore evasisset; sed cùm omni admixtione corporis liberatus, purus et integer esse cœpisset, tum esse sapientem.'"

The second Plate shews us the connexion between the uterine and mammary nerves of the ass—the caudal nerves of the calf—the spinal marrow of the hedgehog—the spinal marrow of the baboon—the spinal marrow of the dog.

The dissections, from which the plates have been delineated, were evidently laborious and, no doubt, correct—the plates themselves are spiritedly got up. The letter-press contains much valuable information on the comparative anatomy of the brain, information of which we shall probably avail ourselves anon. We wish Mr. Swan health to pursue, and public patronage to crown his labours.

---

### NOTICE SUR LE MONESIA.

Mr. Bernard-Derosne, a pharmacien of Paris, has drawn the attention of the profession to this substance, which is obtained from the bark of a tree growing in South America, and to which very wonderful virtues are ascribed. It was introduced into Europe by a French merchant under the name of monesia. It has been employed by several Parisian physicians for two years past, with, it is said, considerable success—especially in scrofulous affections. This bark is of a brown red colour, breaking short and smooth. The extract, as received from America, is nearly black—entirely soluble in water; its taste is at first sweetish, and afterwards astringent, leaving an acrimony long on the tongue. Internally it has been exhibited in bronchitis—hæmoptysis—phthisis—weakness of stomach—diarrhœa—enteritis (?)—leucorrhœa—menorrhagia—blennorrhagia—scrofula—scurvy. Externally in ulcers of various kinds—purulent ophthalmia—hæmorrhoids—ulcers—leucorrhœa.

In chronic bronchitis, the monesia has been administered sometimes alone, sometimes with opium. It appeared to promote expectoration and render the breathing freer. In several cases of hæmoptysis, where the hæmorrhage continued, and resisted other remedies, the monesia succeeded. Although it could not be expected to cure pulmonary consumption, it is said to have been useful in such cases, as a stomachic and expectorant. *Debility of stomach* is one of the maladies for which the monesia has been prescribed, and it is averred, with singular advantage, in doses of 16 grains daily of the extract. Chronic enteritis, as evinced by diarrhœa and pains in the line of the digestive tube, is enumerated among the diseases which have been benefitted by the present remedy. Passing over leucorrhœa, menorrhagia, blennorrhagia and scrofula, we are informed that, in ill-conditioned ulcers, the internal and external administration of monesia has been found very beneficial.

The best internal form of giving it is in pills made of the extract, the dose from twelve to thirty grains daily. For ulcers, the pomade is used—or the powdered extract sprinkled over the sores.

A MEMOIR ON EXTRA-UTERINE GESTATION. By Dr. *Wm. Campbell*, of Queen's College, Edinburgh, &c. &c. Edinburgh, Black; Longman, London.

THE author of this memoir has been long known as a zealous and successful cultivator of obstetric science. During the many years he has devoted himself to this important division of medicine, he has shewn how earnestly he entered into the spirit of its duties both as a teacher and operator. In the parturient chamber, often the poorest and most destitute, when the best resources of the art are most urgently wanted, but most scantily supplied, he has long laboured with highly beneficial results to the afflicted, because he was armed in his endeavours, by the scientific application of means and unwearying humanity. In the lecture-room he has brought the abundant stores of a mind eager in the acquisition of knowledge, long occupied in accumulating, and arranging it, together with the power of communicating it agreeably and effectually to others, thus obeying, and in an important sense, the important injunction, to "do good and communicate." The many who have had the advantage of his public prelections knows how deeply his mind is imbued with the learning and resource of his art, deduced from laborious study and close observation, quickened and polished by critical acuteness; and his published works have proved them to the world.

In preceding publications Dr. Campbell occupied himself chiefly with great practical points in the female economy, endeavouring to shew in what manner and by what means its important maladies might best be combated. The work before us, though practical to a certain extent, is, from the nature of the affection handled, so only in a limited degree. Extra-uterine gestation, though scarcely in strict language deserving the name of disease, is one of the most fearful evils to which woman is exposed. The subject is shrouded in mystery, and is pregnant with interesting matter of a very powerful kind. It is therefore a seductive subject to the inquisitive mind, and highly worthy of investigation; but it must be admitted, after all that has been attempted, that art supplies little in the way of remedy, and judging from the nature of things, never can accomplish much; and that, in defiance of the best efforts of genius, backed by laborious research, it is, and is likely to continue, in a great measure, one of the curiosities—an absorbing and distressing one—of medical literature. Dr. Campbell, in this memoir, has brought together an immense amount of information on the subject. Every available light has been seized for its elucidation, and much pains have been taken so to arrange and convey the various streams, as to exhibit it in the clearest and most intelligible form. From the time of Albucasis to the present day, he has laid under contribution every appliance which could conduce to the object which he wished to accomplish; and the whole shews the great extent of his labors, and how familiar he is with the matter he discusses. As a historical record of extra-uterine gestation, it appears to us complete; and we may safely affirm that, in this respect at least, he has completely exhausted the subject.

We pass over the first section, that on embryology, to notice briefly the contents of the sections on the varieties of extra-uterine gestation, which, as might be expected from the obscurity involving the subject, have led to much doubt and disputation. Three or four distinctions, dependent on difference in the position occupied by the misplaced fœtus, have by many writers of note, such as Breschet, Meyer, Blundell, and Granville, been considered sufficient to express all that is known of the matter; but Dezeimeris, in his love of precision, aided perhaps by love of display, proposes ten or eleven divisions; while G. St. Hilaire admits but two, only one of which is included in the arrangement of the former author. Dr. C. says that "the arrangement of Dezeimeris is unnecessarily minute, and in a practical point of view, useless; since a large majority of the varieties particularised by him cannot be recognised by any diagnostic marks we are acquainted with; and some in the remainder, though there be some symptoms which are very characteristic, yet these even, as must be conceded by every man who has studied the subject, are by no means infallible." He (Dr. C.) proposes the four following divisions of the affection, viz. ovarian, ovario-tubal, tubal, and tubo-uterine. For the adoption of this arrangement he adduces many pathological facts, and states his reasons fully and distinctly, and we are bound to say that the classification appears to us, as far as can be determined, correct, and therefore good. He excludes the ventral variety in the sense generally employed; believing that, in all cases of fœtation not within the uterus, the misplaced product is originally attached to some part of the uterine appendages, not precipitated into the abdominal space, and therefore left in isolated being; and for this belief we think he has offered sufficient arguments. But while we willingly admit this, as well as the accuracy of his arrangement, we are disposed to say to him, though of course with much limitation, what he says to Dezeimeris, "that his arrangement is unnecessarily minute," &c. We do not often ask the scientific enquirer the sordid question cui bono? because good often comes from quarters, and through channels, where it is little expected. Still we fear that little benefit will come from the most elaborate and skilful discrimination of the exact tissue, or portion of tissue, implicated in extra-uterine gestation.

The section occupied with the causes need not detain us; because they are simply conjectural in the present state of our knowledge, and will most probably ever remain so; because such an inquiry can lead to no determinate benefit, and cannot even gratify a reasonable curiosity.

In the section containing an account of the symptoms there is much curious, and some instructive matter, on which, however, our limits forbid us to enter; but we would call particular attention to, and quote from, the section on diagnosis, containing as it does the more peculiar and striking symptoms of the affection. Before, however, giving an extract or two we beg the reader to notice the following, by way of preliminary caution. "A case occurred at Berlin in 1828, in which the section of the abdominal parietes was performed, upon the supposition of a tumor which was felt adjacent to them being an extra-uterine fœtus; instead of which, it proved to be an accumulation of fæcal matter." From Siebold's Journal. This shews the importance of correct diagnosis, especially before we resort to such an operation as ripping up the belly, and may suggest to some the notion, which it will confirm to others, namely, that more than one of the many cases cited under the head of extra-uterine gestation, in this memoir, were abdominal tumors independent of fœtation.

"Although, as in other subjects pertaining to the department of midwifery, much difficulty will occasionally be experienced in establishing a diagnosis, yet assuredly in many instances which may be met with, I should unhesitatingly say, that when the patient is four or five months pregnant, the task is not unsurmountable; but at an earlier stage of gestation, although there may be some striking phenomena, yet we should not be justified in considering them infallible. The nature of the gestation is to be decided, certainly not by a reference to any single symptom, but by a strict analysis of all the most prominent features of any given case. If, after suppression of the catamenia and other phenomena of pregnancy for one or more periods, an individual were to be suddenly seized with uncontrollably acute pains in either iliac region, even antecedently to the period of quickening, accompanied by a well-defined swelling at a corresponding point, sanguineo-mucous discharges per vaginam; frequent desire for, and pain attending micturition, tenesmus, with a sense of fainting, such ailments ought certainly to warrant a practitioner in suspecting an ovarian or tubal pregnancy.*

* "Ce chirurgien célèbre dit avoir vu deux femmes enceintes de quatre mois,

Were the uterus found in an elevated position in the pelvis, and besides this organ, an additional body detected in the same cavity, such a discovery might certainly be viewed as a corroboration of our suspicions.

When, after the presence of fœtal movement cannot be questioned, the cervix uteri is found directed towards the pubis, so much elevated in the brim, that it can be felt with difficulty, or that it cannot be reached at all, there need, generally speaking, be very little doubt as to the presence of an extra-uterine gestation. An additional support to this diagnostic is an undeveloped state of the cervix, and of the corpus uteri when it can be felt. Another point which deserves particular attention is, whether the abdominal tumefaction was observed to have commenced at one side, and whether fœtal movement was remarked to have for a time occupied a corresponding point. When gestation is far advanced, and the adventitious cyst occupies a large share of the abdominal cavity, these diagnostics cannot be relied on, though of great value at an earlier stage. In connexion with the foregoing remarks, we must not overlook the importance of occasional paroxysms of acute pains, in different regions of the abdomen, as very characteristic of extra-uterine gestation. Of all the phenomena which may be particularized, none has commanded attention so frequently, as the altered position and undeveloped condition of the uterus, as may be observed in the histories of many of the cases which have been offered in illustration.

From the foregoing illustrations it may be observed, that in occasional instances extra-uterine gestation has been confounded with retroversion of the uterus; but a little reflection must satisfy the practitioner, that there is a marked difference betwixt the symptoms of each. In retroversion the patient is suddenly seized with almost total inability to void urine, and with tenesmus, but inability to evacuate the rectum, and these symptoms are invariably present; while in extra-uterine gestation, although there is frequent desire, and much pain in performing these functions, yet they are never obstructed, as in retroversion; and, moreover, these complaints supervene in paroxysms, or they are present only in occasional instances. In regard to the malposition of the uterus, the only symptom in which these derangements strikingly resemble each other, this likewise is but occasionally felt in extra-uterine gestation, while it always happens in retroversion. We have, moreover to remember, in reference to this last point, *first*, that the pelvic cavity, except in some very rare instances, is not at any time so completely occupied by the tumor of an extra-uterine gestation, as by the uterus in a state of retroversion; and, *secondly*, that the patient can freely evacuate the bladder, when the pelvic tumor is pushed towards the sacrum."

We omit what the Doctor has written in the three subsequent paragraphs on diagnosis, because we do not see their application to the subject in hand; because we do not intend here to enter on the *questio vexata* of the *viability* of children; and because we more than question the taste or utility of introducing the Rev. Dr.

---

chez lesquelles rien n'indiquait ce que leur etat avait d'extraordinaire, si ce n'est des tiraillements frequents dans l'abdomen et la tumefaction irrégulière du ventre qui se portait d'un côté seulement. Elles eprouvèrent les symptômes suivants lors de la rupture des trompes: elles furent attaquées inopinement de douleurs extrémement vives qui durerent deux ou trois heures; une douleur plus forte que les autres fut suivie d'un calme parfait; le ventre s'affaissa et fut comme aplati; une chaleur égale et douce se répandit dans la cavité abdominale; la peau se decolora, il survint des syncopes presques continuelles; le pouls s'affaiblit et se concentra; une sueur froide se repandit sur toute la surface du corps, et les malades expirèrent; Sebatier, Med. Operat. I[ere] edition, t. i. p. 343."

Chalmers' and the Kinghorn cause into a memoir like this. What he says of ourselves we very freely forgive. We have lived too long amid the storms of the ocean to be much moved by the ripple of a river, or the heavings of a Sylvan lake. In the occasional conflicts in which we have been engaged, and which we could not always, however we might desire it, avoid, we have used the weapons in our hands as gently as was compatible with self-defence, and our championship, though self-constituted, of the truth. When in dealing with distinguished members of the profession, we have felt it our reluctant duty, it might be in error on our part, to find fault, we have done so in kindly feeling and courteous language, certainly with no desire to irritate or provoke; on the contrary, we would, if we could conscientiously, deal only in praise, and eschew altogether, in our intercourse with the genus irritabile vatum, the ungracious and unacceptable task of censuring; but in that case, criticism, whatever it be worth, would be at an end. Having skipped the three paragraphs in question, we give the following and concluding one, from the chapter on diagnosis.

"As we might be encouraged to have recourse to gastrotomy by the prospect of emancipating a living child, this essential point must be determined chiefly by the careful application of the stethoscope. The declarations of the parent, that she is conscious of fœtal movement, cannot be relied on; since it must be well known to practitioners, that females often persist in stating that they have been sensible of the motions of the child to within a few hours, or indeed minutes, of its birth; while, from the degree of decomposition which it then exhibited, no doubt could be entertained that life must have become extinct several weeks previously. Violent fœtal struggles, preceded or followed by rigors and expulsive efforts, and thereafter total cessation of motion, are proofs of the extinction of fœtal life that will rarely deceive a practitioner. When these are followed by diminished tumidity of the abdomen, the secretion of milk in the mammæ, and the return of the catamenia, another conclusion may be drawn with equal certainty, viz. that the gestation is extra-uterine."

We can no further enter on the sections on prognosis, termination, and pathology, than to say, that they will well repay perusal, and shall conclude our notice of this very interesting publication, by glancing at the chapter on treatment.

This, our author considers under four heads:—*first,* the precautions required to retard or prevent laceration of the cyst; *secondly,* the measures necessary to moderate the hæmorrhage after laceration; *thirdly,* the management after the extinction of fœtal life; *fourthly,* the steps to be pursued for the emancipation of the fœtus. On all these points the observations of Dr. Campbell are judicious, though unfortunately, from the nature of the subject, too little satisfactory in a curative view, to induce us to follow them.

---

PRACTICAL OBSERVATIONS ON ABORTION. By *J. S. Streeter,* Member of the Royal College of Surgeons, President of the Westminster Medical Society, Honorary Member of the Physical Society, Guy's Hospital, &c. With Plates and Wood-cuts. 8vo. pp. 70. London: Sherwood, Gilbert, and Piper, 1840.

MR. Streeter is a gentleman engaged in an extensive general practice, and it reflects no small degree of credit on him, that, harassed as he must be with calls upon his time and his attention, he should cultivate the *science* of his profession with such ardour and success. Those who enjoy the pleasure of his acquaintance can best speak to that enthusiasm which he displays, and which alone can conduct to anything great or honourable in medicine.

Mr. Streeter informs us that, being occupied with the study of the development of the nervous system, he was naturally led to examine such ova, passed by abortion, as came under his notice as an accoucheur. "I experienced," he says, "little difficulty in procuring fœtuses which had arrived at the middle periods, but observation soon convinced me that perfect and well-formed embryos were rarely expelled in miscarriage till after the third month had been completed; indeed, in most instances, before that time the ovum when passed entire was found on being opened to contain no fœtus, or one in a blighted or decayed condition; and I noticed that, where such ova were thrown off by abortion, my patients had resisted the usual means of prevention, that they often recovered rapidly, and showed little or no tendency to miscarriage in subsequent pregnancies." Many interesting cases of threatened or of actual miscarriage having occurred to Mr. Streeter, he was induced to review the whole subject, and ultimately to lay the result of his inquiries before the Westminster Medical Society, in the shape of a distinct essay. The present work is that essay in another form, and with some slight additions, and the main object of the author in addressing both the Society and the profession may be gleaned from the following passage.

"The practical points which I was anxious to enforce on the members of the Society were a more exact distinction between the premonitory and the essential symptoms of miscarriage, the impropriety of the use of opium, and the advantage of that of ergot during the presence of the latter, and the disadvantages of a casual or intentional rupture of the membranes during the process of abortion. Lastly, I wished to direct attention to the impossibility of laying down rules for the prevention of miscarriage in any subsequent pregnancy, without first determining, from accurate examination and competent knowledge of embryology, the normal or pathological conditions of the aborted ovum, and hereby arriving at the real cause of the miscarriage, instead of indolently and vaguely assigning it to the last accompanying circumstance."

The work consists of five chapters, the first devoted to some general observations—the second to the structure of the ovum—the third to the nature, symptoms, and causes of abortion—the fourth to the treatment of abortion—and the fifth to the prevention of abortion in any future pregnancy.

1. In his general observations, Mr. Streeter defines what he means by abortion or miscarriage (he employs the terms synonimously). He considers it, with Dr. Granville, as the premature extrusion of the ovum before its fœtus is capable of maintaining life, independent of the parent,—if it is so capable, it is premature labour and not abortion. He eloquently denounces the absurd distinction still preserved in our criminal code, between the procuring abortion *before* and *after* the period of quickening, and the staying of execution of pregnant criminals, only *after* that event has taken place.

2. The chapter on the structure of the ovum affords ample evidence of the industry, and of the judgment of our author. But it is not necessary for us to follow him in his embryological sketch.

3. Mr. Streeter remarks, that abortion may occur from the ovum not forming its normal attachment to the uterus after entering that organ in consequence of a defective formation of the decidual lining. More commonly, however, miscarriage does not occur until after the attachment of the ovum, when it usually consists:—

1st,—In the destruction of that membranous and vascular attachment which the ovum normally forms, by means of the uterine envelopes and the placenta, to the interior of the uterus.

2nd,—In the dilatation and opening of the neck and mouth of that organ, and,

Lastly,—In the extrusion of the unaffixed or detached ovum.

These stages, however, occur, in general, more or less simultaneously. Mr. Streeter goes on to observe that the exact nature of the "utero-ovine" attachment varies with the period of pregnancy. "At the commencement of gestation," he says, "when the connexion is entirely decidual, we might easily persuade ourselves that the union between the ovum and the uterus was weak, and that accidental shocks would more easily destroy it than after the formation of the placenta had occurred; but the contiary seems to be the fact. Perfectly normal ova are very rarely thrown out of the uterus by abortion, during the earlier weeks of utero-gestation; they are, in fact, seldom met with before the middle, and are most frequent of all in the latter months of gestation. This circumstance is of itself sufficient to show that the natural connexion between a healthy ovum and the uterus in the early stages of pregnancy is ample, and not easily broken; but the same experience also shows that, when the ovum is defective and the placenta not regularly formed, or becomes diseased, slight accidents may, and often do, lead to its complete separation and subsequent extrusion. When ova so thrown off are carefully opened, the fœtus will be found imperfectly evolved, withered, or entirely wanting."

The agents of separation of the "utero-ovine" attachment are, 1st, muscular contraction of the uterus; 2ndly, an increased arterial action, which leads to extravasation of blood between the ovum and uterus. The symptoms therefore which essentially attend the process of abortion are two, namely, uterine pains of two kinds,—the pains of dilatation and expulsion; and the more formidable one of hæmorrhage.

"Where the separation of the ovum results from vascular excitement of the uterus I believe a stage of premonition will have usually existed, too often indeed disregarded from the trivial inconvenience it occasions while present. It is marked, however, by sensations of weight, uneasiness and fulness about the pubic, inguinal, and lumbar regions; by the sympathetic pains in fact which accompany congestions of the pelvic viscera, and these are not unfrequently attended by white or coloured discharge from the vagina, and by irritation of the alvine or urinary organs. There is a teazing diarrhœa or frequent and unusually pressing calls to relieve the bladder—evidences of an increase of sensibility in these organs to their ordinary stimuli, and which encreased sensibility is likely to extend to the uterus itself and set up expulsive action there.

Where the death of the embryo occurs in the early period of gestation, the essential symptoms of miscarriage are often preceded by a sudden and premature cessation of the customary morning sickness. In later periods a softness of the mammæ and a recession of the areolar changes when present, in many instances announce the approach of miscarriage. Where the placenta is withered as well as the fœtus, there is often superadded to these an offensive mucous discharge from the vagina for some time before the process of abortion actually begins. It is right, however, to state that the presence or absence of any of these symptoms cannot be looked upon as a certain diagnostic sign of the death or life of the fœtus; but collectively their presence amounts to a very strong presumption that the embryo has ceased to live, and that all attempts to prevent expulsion will prove unsuccessful, if not absolutely injurious, to the health and well-doing of the mother."

Mr. Streeter remarks, that the causes of abortion are all resolvable into three great classes:—

1st,—An imperfect or abnormal formation of the ovum. This may take place either in the fœtus or its membranes; or in the decidua and placenta, those structures which fasten the ovum to the uterus and finally connect the embryonic and the maternal systems.

2ndly,—Morbid states, functional or organic, of the uterus and its appendages.

3rdly,—Morbid states generally of the constitution of the mother.

He believes that imperfect or abnormal formation of some part of the ovum, or of its connecting media is by far the most frequent cause of early miscarriages. He illustrates this by two figures from preparations in his own collection. The death of the fœtus, however, he observes, does not by any means lead to the immediate expulsion of the ovum. The latter does not usually happen till certain changes have occurred in the structures of the utero-ovine attachment consequent on that event, in the uterine envelopes principally during the earlier, in the placenta more exclusively at the later, periods of gestation. The knowledge of this fact is most important, as it may, under certain circumstances, involve the reputation of the mother. It is also intimately connected with the pathology of those diseased ova which ordinarily receive the name of moles. In cases of twin conception, where the ova are not adherent, one fœtus may die early and be expelled prematurely, while the other may be carried to the full term and be born alive; but if the placentæ, as more commonly happens, are united together, the dead fœtus may lead to the premature expulsion of both, or it may be retained till the other is born alive.

Mr. Streeter does not venture on details of the pathological conditions of the ovum in general. But he makes a remark which we shall quote:—

"In examining aborted ova, it is not uncommon, on slitting up the chorion, to find two vesicles intimately connected and communicating, filled with fluid which in one instance remained transparent, in another threw down a large quantity of deposit when preserved in spirit. On mentioning this circumstance to Dr. Sharpey, he remarked, that it was not correct to assume that every vesicle found within a chorion was necessarily an amnion, for it might be a yolk vesicle. I am therefore led to regard the vesicle met with in one of the Velpeau's three early ova, in the one marked No. 2525.5, in Guy's Hospital Museum, and in several in that of University College, where there are vesicles contained within the chorion, not as instances of the presence of an amnion without a fœtus, but as degenerated yolk-sacks. In an ovum in the collection of Mr. Wells, where the vesicle is double and intercommunicating, and also in a similar one in the possession of Mr. J. Gregory Smith, I consider that the germinal membrane had commenced its evolution, that it had proceeded so far as to form its cephalic and caudal folds, and that both yolk-sac and embryo had undergone degeneration, and become mere vesicles."

He passes to those causes inducing abortion which occur from disease of the uterus or its appendages—and alludes to irritability of the muscular fibres of the uterus, however excited—apoplexies of the membranes or extravasations of blood between the uterus and ovum, and those effusions between the reflexa and chorion which gradually dissect up the reflexa from the chorion and form solid and tubercular-looking coagula—and those diseases of the interior of the uterus which lead to non-formation or imperfection of the uterine and ovine deciduæ, or after their formation to acute, subacute, or chronic inflammation of its lining membrane, especially of that part where the decidua serotina is evolved, and which participates in the formation of the placenta, the part which furnishes indeed what may be termed the uterine element of that organ. He conceives that we must turn for an intelligible explanation of hypertrophied or coriaceous states of the uterine membranes, for the origin of those pseudo-membranous formations external to the chorion which often cause so much perplexity in making out the real normal envelopes of the ovum, and for the production of those degenerated and solid ova which are termed moles. Lastly, we must refer to defective or morbid action of the same part for an explanation of those imperfections of the placenta which are so frequently found associated with fœtuses emaciated to the greatest possible extent.

In referring to the constitutional causes of abortion, Mr. Streeter says—"I believe that so long as the utero-ovine connexion remains healthy, and the excitability of the uterus is not exalted above its natural standard, that abortion is not produced by constitutional disease, by organic disease of remote organs, or by general states of plethora or debility. Let me not, however, be misunderstood as underrating the influence of constitutional disturbance in increasing the nervous susceptibility, or in exciting latent predisposition to disease in the uterine as well as in other organs. All that I mean to assert is, that general causes exercise no more immediate or specific influence over the uterus than over other important organs, as for instance over the heart, the stomach, and the brain. Some of the diseases enumerated, the exanthemata, for instance, may extend to the fœtus and destroy it, and so cause abortion, but unless they first produce its death, abortion does not as a general rule ensue."

4. The subject of *Treatment* occupies the fourth chapter.

Mr. Streeter doubts the propriety of employing opium in the earlier weeks and months of pregnancy—he seldom has recourse to it where abortion is threatened before the third month of pregnancy is completed.

"When however in addition to uterine pain these early cases are attended with any hæmorrhage, I lay it down as a canon of treatment that the practitioner ought not to employ opium, or any drug that has the property of suspending or lessening uterine contraction. The results of enquiries show that miscarriage in almost all instances arises from causes over which, when its *essential symptoms* have set in, the accoucheur has lost all control. Fœtal or uterine disease or imperfection has accomplished its work of destruction, and the blighted embryonic thing must now be cast out from the womb. If the ovum is not so blighted, rest in the recumbent or horizontal position, mental quietude, the abstaining from stimulating articles of food, the clearing out of the bowels by a simple enema or mild purgative, the administration of common salines, such as the citrate of potass with small doses of digitalis and hyoscyamus, will suffice to allay the symptoms. When however the ovum is blighted, it must necessarily be thrown off by miscarriage, and the suspending the uterine pains by the administration of opium only retards, and in too many instances disarranges, that process altogether. If therefore hæmorrhage continues or recurs after a few hours' trial of the above means, it seems to me that it then becomes a duty to ascertain by vaginal examination whether the os uteri has begun to dilate or not. If dilatation of this part has commenced, or if the hæmorrhage steadily encreases, even before it becomes profuse or tells upon the constitution, the ergot of rye should be freely and fearlessly employed in larger or smaller doses, and given at longer or shorter intervals according to the effects which it produces. The accoucheur should from time to time as gently as possible ascertain the extent of dilatation of the os uteri, remove all clots from the vagina, and dislodge the ovum if, as sometimes happens, it is merely adhering to the os uteri and keeping up hæmorrhage by the irritation which it occasions there."

Keeping the room of a moderate temperature—the horizontal posture—cold drinks—light food—stimuli if syncope impends—and, should the symptoms become alarming, pressure on the abdominal aorta and plugging of the vagina. Permanent contraction of the uterus can often be brought on and may nearly always be sustained by regularly-repeated doses of the ergot of rye, and the use of cold and astringent injections into the rectum. When all other means fail, the sole remaining resource is transfusion.

Should the membranes rupture when the cervix is only partially opened, or before the separation of the ovum from the fundus and body of the uterus is complete, the liquor amnii is expelled, and if small the fœtus also, and the partially separated membranes and placenta alone remain. If the separation from the fundus is complete, the membranes are often partially or wholly everted, and may be felt hanging out from and fringing the os uteri. The process of extrusion is now long retarded, and effected at last by a process resembling putrefaction. If hæmorrhage continues, and the vagina is lax, and the cervix uteri sufficiently dilated, the introduction of a finger a little way into the uterus will often enable the practitioner to dislodge the ruptured ovum, but if this cannot be done by gentle means, the use of the plug is the preferable proceeding. After the fifth month, the manual interference employed for hæmorrhage at the full term may often be resorted to with advantage.

Frequently, after the rupture of the membranes the hæmorrhage ceases altogether, or becomes so trifling as not to influence the constitution, and the pains subside or entirely disappear. The most advisable practice when this happens, is to try and reach the inverted membranes, and to gently dislodge them with the finger; but if they tear, or the attempt at removal produces much pain, it is proper to desist; as long as there is no hæmorrhage it is better to wait, to watch the patient carefully, and just before or as soon as putrefaction sets in, to frequently wash out the vagina and uterus with injections of tepid water. The patient is, at the same time, to be supported, and Mr. S. has never known mischief happen.

It has been recommended in these cases to hook out the placenta and the membranes with a small wire crotchet, or to seize the protruding parts with a pair of polypus forceps, and so extract them; but Mr. Streeter would prefer employing the continued injection of a stream of tepid water by Read's syringe.

5. The prevention of abortion in any future pregnancy occupies the last chapter of the work. Mr. Streeter holds it to be absolutely essential, that the ovum which has been expelled, whether it has come away entire or in fragments, should along with its envelopes be submitted to a minute and careful examination. This is most conveniently done after the ovum has been suspended for a few hours in a large vessel of water, so as to allow the colouring matter of the blood to disengage itself and subside to the bottom; and indeed the whole examination is most distinctly and satisfactorily conducted under water. This examination seldom fails to elicit, whether the ovum has perished from inherent imperfection, or from the fault of the uterine envelopes, or placenta. A most important practical distinction.

"When examination shows, as it commonly will during the first three months of gestation, that abortion has arisen from inherent imperfection in the ovum uncomplicated with uterine disease or hæmorrhage, the after-treatment required is of the most simple description: the lochial discharge in general quickly subsides, the catamenial secretion is re-established in due time, and impregnation usually takes place again at no very distant period. According to my experience females seldom have a long succession of imperfect ova without an intermixture of perfect ones, and unless the uterus is itself in fault, the perfect ova are retained till the full term of gestation is accomplished."

In such a case, the female is to be managed in a future pregnancy, just as if she had never miscarried.

But when there are effusions of blood or traces of inflammatory disease in those membranes which are derived from the uterus or in the placenta, the patient must at once be placed under appropriate treatment. Bland injections should be thrown into the vagina, and such constitutional management entered on as seems adapted to the case. The morbid state of the interior of the uterus is however seldom completely removed without the strictest attention to diet, change of air, and a long and uninterrupted cessation of cohabitation.

When such a female again becomes pregnant, the utmost care is requisite. She must submit to an antiphlogistic regimen, the bowels must be carefully regulated, and bleeding followed by an opiate should be occasionally employed as a measure of precaution, and especially whenever symptoms of general or uterine plethora show themselves. Bodily quietude should be preserved, but not carried so far as to bring on mental or constitutional irritability. Gentle exercise—the recumbent posture, however, about the expected period of miscarriage—attention to the pelvic viscera—abstinence from cohabitation.

"Examination in some few instances shows no appreciable disease or imperfection of the ovum or its membranes, and then the miscarriage must be assigned to a morbid excitability of the uterus. Morbid irritability of the uterus is, I believe, seldom the sole or immediate cause of abortion till the utero-ovine connexion has become almost wholly placental, and this is not the case till after the fifth month of gestation has commenced. It is indeed the most frequent cause of labour coming on prematurely after women have experienced accidents, or sudden and severe mental emotion; but its presence in the earlier months predisposes more to irregular determination and congestion of blood, and consequent effusion on the membranes, than to any irregular and expulsive action of the muscular fibres of the uterus."

These cases are troublesome, miscarriage often occurring in a series of successive pregnancies, till the tone of the uterine fibres has been lowered by distention, and their susceptibility to contraction has been thereby lessened. Change of air—tepid or cold sea-water baths—suitable tonics, &c. are requisite. If pregnancy takes place before the susceptible state of uterus is removed, the chief, if not the only means upon which reliance can be placed for preventing a premature expulsion of the fœtus, will be the timely and persevering use of opium, exhibited in the form that agrees best.

Some other mixed cases not unfrequently present themselves in practice. An inherently defective ovum, for instance, is found accompanied with either an irritable or an inflammatory condition of the uterus; or again, an inflammatory state of the uterus may coexist with an irritable one. Such mixed cases demand mixed treatment. This should partake more largely of the character of that which is in use for subduing inflammation on the one hand, or for allaying irritability on the other, according as the symptoms and appearances of the aborted ovum proclaim the one or the other state of the uterine system to predominate. Early and moderate bleedings, alternated with opium, abstinence from cohabitation, attention to the health, &c. must be had recourse to.

Mr. Streeter concludes by observing that, "the practical value which I have attached to the information derived from the examination of aborted ova may by many practitioners be thought over-rated. They have perhaps regarded the pathology of the ovum as a subject altogether more curious than useful, but when they have adopted the indications which it affords, they will soon recognize its worth in practice."

We think he deserves much credit for the manner in which he has carried out this sound pathological view.

---

THE EYE: A TREATISE ON THE ART OF PRESERVING THIS ORGAN IN A HEALTHY CONDITION, AND OF IMPROVING THE SIGHT; TO WHICH IS PREFIXED A VIEW OF THE ANATOMY AND PHYSIOLOGY OF THE EYE; WITH OBSERVATIONS ON ITS EXPRESSION AS INDICATIVE OF THE CHARACTER AND EMOTIONS ON THE MIND. By *J. Ch. August. Franz*, M.D. &c. &c. London: J. Churchill. 1839.

Dr. Franz informs us, in a short Preface, that his chief object has been to point out the principles by which the organ of sight is preserved in a sound and healthy condition. He has, accordingly commenced with the age of childhood, and, proceeding through the successive periods of life, he has considered, the circumstances peculiar to each period, and called attention to whatever is calculated to prove either beneficial or injurious to the eye. Dr. Franz apologises for writing in a tongue which is not his native one; but such excuses are unnecessary, for Dr. Franz writes well.

The work consists of two Parts. The *first* embraces the anatomy and physiology of the eye—the importance and dignity of this organ—and its expression as indicative of character. The fourth chapter, that on the expression of the eye as indicative of character, is very elaborately worked, and might form a pendent to Lavater. The enumeration of its contents will give our readers a notion of the pains expended on it—pains characteristic of the laborious nation of the author.

It comprises—observations on the look and its principal differences. Under the head of expression of the eye with reference to the quality and condition of the mind, we have, indication of the innate constitution of the mind—the eye of the man of powerful understanding—the eye of the man of impassioned feeling—the eye of the man of energetic volition—the eye of the man of talent, and creative genius—the eye of the man of limited capacity—indication of the habitual disposition of the mind—the moral condition—the eye in virtue and piety—the eye in innocence—the immoral condition—the eye in immorality—the eye in vice—indication of the emotions of the mind—affections—the eye in love—the eye in unsuccessful love—the eye in hatred—the eye in joy—the eye in sorrow—the eye in hope—the eye in despair—the eye in fear—passions—the eye in terror and horror—the eye in anger and revenge—the eye in envy and jealousy. Then come sections on the expression of the eye at the different periods of life—expression of the eye as influenced by difference of sex—expression of the eye in reference to the moral and intellectual condition of nations, and lastly, practical observations on the expression of the eye, especially of the look. Study of the look important to metaphysicians and moralists—study of the look important to members of the legal profession.

We have glanced over these descriptions of the various expressions of the eye, and we must confess that they are rather beyond us. Not that we mean to refuse to the eye the power of expression in a high degree—it is the diagnosis that staggers us.

There is a passage, by the way, that may give some hints to the New Police, in these murderous days, and, if duly studied by the Inspectors, may help them to retrieve their character.

" Lastly," says Dr. Franz, " the study and knowledge of the indicative character of the eye is of the highest importance in the inquests and post-mortem examinations held on the bodies of persons who have been murdered,—if the person who has committed the crime, or who has been concerned in it, is present in the house or apartment where the examination is carried on.

If in such cases an individual is observed to look round in an anxious, uneasy, and confused manner, at one moment with a sad and dejected air, and at another with affected unconcern, or forced cheerfulness; if there is an unusual bustling activity and restlessness in his manner; it may in general be fairly suspected that he is either the actual perpetrator of the deed, or has been at least an accessary, and is consequently arraigned at the bar of conscience. The eyes here always give the first intimations of guilt. The psychologist must certainly observe with especial care and attention the eyes, as well as the countenance and behaviour of this person, and before he forms a determined opinion, he should endeavour to ascertain by close inquiry the following particulars :—

1. What is the character of the person in question, both as to his moral conduct, general habits, and mode of life?

2. What is his habitual state of feeling; whether it was previously different from that which he evinced during the inquest and examination?

3. Whether he has on all occasions manifested a great degree of horror at the sight of a dead body?

4. What is his general demeanour; and whether it is reserved, anxious, and timid in the presence of strangers, and especially of officers of justice?

5. Whether he has been nearly and intimately connected, either by the ties of kindred, or in any other way, with the unhappy person whose death has been the subject of investigation?"

Thieves and murderers are so hardened in this country, that we fear they will laugh at this physiological cross-examination, and be too apt to consider it "all my eye."

The *Second Part* is devoted to the Art of Preserving the Eye in a Healthy Condition, and of Improving the Sight: Management of Ophthalmic Diseases in their Incipient Stage. It consists of six Chapters—the first, on the Eye in Infancy—the second, the Eye in Childhood—the third, the Eye in Youth—the fourth, the Eye in Manhood—the fifth, the Eye in Old Age—the sixth, General Regimen with reference to the Eye.

In the Chapter on the Eye in Youth, Dr. Franz protests, for the eye's sake, and on its account, against tight-lacing, stiff cravats, and tobacco.

With reference to the two first, he observes that, such imprudent confinement of the neck, or of the body generally, may give occasion for the development of dangerous inflammation of the eyes, myopy, or actual weakness of sight; or may even lay the foundation, though the progress may be slow, and the disease may uot manifest itself for years, of cataract, or of amaurosis. So that not only crooked spines, bad lungs, and bad livers, but bad eyes, *flow* from tight-lacing.

We all of us would wish to keep our eyes and our eyesight as long as possible, and we must do Dr. Franz the justice to say that he has tried his best to preserve them for us. At every age, from babyhood to the "last sad stage of all," we have minute instructions for the preservation of these valuable organs.

As all medical men are consulted, at times, on the use and the selection of glasses, we are tempted to glance at Dr. Franz's directions for their use. He observes:—

The ordinary glasses are made of plate glass, which is not always sufficiently hard, and often not pure enough; but the glasses which are made of Brazil pebbles deserve the preference. When the pebble is properly cut, and the glasses are finished with care, and are well suited to the condition of the eyes, they have a beneficial influence upon the organ of sight. They are, it is true, very expensive, but are at the same time very durable, and persons who can afford to pay for them ought not to grudge their higher price. The glass is usually colourless, sometimes coloured. Whatever material employed, the principal requisite in all cases is, that it should be as hard as possible, perfectly free from spots, striæ, air-bubbles, &c. and without mixture of colours; and in the next place, that the greatest accuracy and care should be observed in grinding and polishing the glasses. The grinding is performed upon moulds or cups, which constitute a segment of a sphere, and thus give the form to the surface of the glasses. Convex glasses are named according to their focal length, and the concave are numbered in indication of their degree of concavity. No 1, for instance, on a concave glass, marks the smallest degree of concavity; No. 2, a somewhat higher, and so forth.*

Glasses may be plane and non-refracting, or bi-convex, or bi-concave, or plano-convex, or plano-concave.

---

* The numbering of glasses is unfortunately not everywhere according to the same standard. The glass which with one optician is No. 1, is by another marked No. 2. If, therefore, a person has broken or lost the glass which he had in use, say No. 2, for instance, he must not ask for and purchase at random another No. 2, but must first by carefully trying it ascertain whether the new one be suitable.

"In using the glasses hitherto described, it is necessary to bring the head into the direction of the object which we wish to view. To remedy this inconvenience glasses have been invented, in the employment of which it is only required to turn the eyes towards the object, without turning the head at the same time; these are:

The periscopic glasses, as they are termed, the outer surface of which, or that which is turned away from the eye, is convex and the inner concave; they are particularly recommended by Dr. Wollaston.

There are also glasses with cylindrical surfaces. Independently however, of the circumstance, that glasses of this, as well as of the preceding description, being difficult to make, are seldom quite perfect, they have among other disadvantages this in particular, that objects are seen somewhat altered from their true form, and are less distinctly recognised than with the other kinds of glasses. Neither of these kinds therefore is to be preferred to those previously mentioned.

Every glass, of whatever form its surface may be, ought to be large enough to cover the eye perfectly. The oval form is better adapted for spectacles, the round for hand-glasses."

The frame, or mounting of glasses, he continues, is not immaterial. In order to avoid false light, it must be dark, and not polished; it should be easy, and somewhat flexible, that it may not become incommodious when used for a long time. The most suitable material for the frame is either unpolished blue steel, horn, or tortoise-shell. The old shape, in which the spectacles were pressed and held fast upon the nose by means of a narrow arch, was objectionable. The kind now in common use is far better; these are fastened on the head by means of two long jointed sides. In this manner the glasses are kept in a fixed position before the eyes, and if the bridge over the nose be curved at one side only, the same glass must always be applied to the same eye, as they cannot then be used inverted; this is of importance when there is a difference in the sight of the two eyes. The frame of spectacles must be so contrived, or the spectacles at least fixed before the eyes in such a manner, that the axis of the eye may coincide with that of the glass. To see remote objects therefore the glasses must stand more perpendicularly before the eyes, and to see near objects they must be inclined somewhat forwards. The glasses moreover ought not to lie in the same plane; their position should observe the direction of an obtuse angle of such degree that the axes of the eyes, which of course converge more in reading and writing than in viewing distant objects, may exactly coincide with the axes of the glasses. If these rules are neglected, the spectacles do not represent objects clearly and distinctly; they occasion an unpleasant sensation in the eyes, and even giddiness and head-ache; and contribute likewise to produce the squinting and fixed look which is frequently observed when the spectacles are taken off.

Dr. Franz inquires, when eye-glasses should be first employed, how chosen, and in what manner used. He properly reprobates the use of an eye-glass for fashion's sake; it *is* ridiculous. He thinks that the frequent use of concave glasses by young people is not always the consequence of myopy, but more often the cause of this defect of vision. Persons who do not think their sight good enough for very distant objects, should by no means have immediate recourse to eye-glasses, as myopy is frequently induced by this practice.

In the first degree of myopy, when, for instance, an ordinary-sized type can be read at the distance of ten inches, weak plano-concave glasses in the form of double hand-glasses, better however in that of spectacles, may be used, but for viewing distant objects only. A single glass is objectionable. In a moderate degree of myopy, the exertion of the eyes, and even of the body, may be much relieved during such occupations with near objects as require an extensive field of view, by very weak concave spectacles. For distant objects they must be stronger. When the degree of myopy is such, that even large objects at several paces distance cannot be recognised without difficulty, and the eyelids are much drawn together in looking at them; when small objects, or the book, in reading, must be brought very near to the eye, perhaps as near as three or four inches; when, consequently, use is made only of one eye to view the object, because on account of its proximity the axes of vision can scarcely be united upon it: spectacles are in such cases indispensable. One of the principal rules, however, to be observed in all cases of myopy is, to have recourse to eye-glasses rather somewhat *late* than too early.

"With respect to the selection of glasses, let the choice never fall upon a single concave glass, and let it be rather upon a pair of spectacles than double hand-glasses; because glasses in the form of spectacles remain at rest and in a proper position before the eyes. Let the lowest possible number that will answer the desired purpose be chosen. If the glasses enable the individual to see distant objects clearly, or if they relieve the exertion of the eyes when employed upon near objects, they are suitable, and he ought to be satisfied with them. If he choose a higher number, he will certainly have the advantage of seeing distant objects more distinctly, but the sight is equally tried whether glasses that are too strong, or no glasses at all are made use of; in either case the myopy increases. The person desiring the aid of spectacles will always do well to apply to an experienced and intelligent optician, and to communicate the four following particulars.

1. What degree of myopy exists, that is to say, at what distance he can still see clearly; and whether it is the same in each eye. Both eyes must accordingly be tried beforehand, in the manner explained at p. 170, first together, and then each eye separately; for the latter purpose one eye should be covered, while the experiment is made with the other.

2. At what distance he wishes to be enabled to see clearly. For reading or writing, twelve to fifteen inches may be assumed as a proper distance.

3. Whether the spectacles are required only for some particular occupation, and if so, of what nature the occupation is, or whether they are intended to be worn constantly.

4. Whether he intends to use the spectacles chiefly in the day-time, or in artificial light."

Spectacles should be tried for some time before they are finally selected. They should be used as moderately as possible, and the myopy not unfrequently disappears.

"After having used the glasses first chosen for some years, if the individual is not so perfectly satisfied with them as at first, the myopy has either increased or diminished. It is accordingly very injudicious to procure without farther consideration glasses of higher power, as is unfortunately the usual practice in such cases. The near-sighted person should first endeavour to ascertain the present condition of his eyes by trials of the sight with the naked eye. If by comparing the result of the present trial with that which was obtained on first having recourse to glasses, he find that the myopy has diminished, the glasses hitherto used are either to be exchanged for weaker, or to be entirely laid aside. But if he find that it has increased, it is more advisable to retain still the same glasses, and to exercise his eyes in looking at distant objects, observing at the same time the general rules before-mentioned, than to exchange them for stronger. Glasses of higher power may however be necessary, if the myopy, owing to any unavoidable injury, has so much increased, that the occupation causes exertion and fatigue to the eyes, even when proper glasses have been selected in the first instance. But it is always better to refrain as much as possible from the exchange of glasses; for persons, who without consideration are continually having recourse to glasses of stronger and stronger power, run the hazard of being at last unable to procure any glasses which can afford them satisfaction, and consequently of becoming, as it were, entirely blind. Glasses which are employed chiefly for particular occupations with near and fine objects, ought not to be used for distant objects. In a very high degree of myopy, it is well to possess one pair of spectacles for remote, and another for near objects, and even a third for occupations in the evening."

*Far sighted* persons should seek the aid of glasses too early rather than too late, a rule the reverse of that which applies to the near-sighted. They too should use spectacles, begin with a weak power, and institute a careful trial previously to choosing. With the advance of age stronger glasses are necessary, but they should be charily adopted.

We must here conclude our notice of this little work, which we recommend to the public, perhaps to the profession, as well calculated, if its precepts are acted on, to preserve the eyes of its readers. Of Dr. Franz we entertain a high opinion, and he may be assured that his English brethren welcome his appearance amongst them as an author, as well as a physician.

---

THE INTIMATE STRUCTURE OF SECRETING GLANDS. By *John Müller*, M.D., Professor of Anatomy and Physiology in the University of Berlin. With the subsequent Discoveries of other Authors. By *Samuel Solly*, F.R.S., Lecturer on Physiology, and on Comparative Anatomy, at St. Thomas's Hospital, &c. London: Joseph Butler. 1840.

Mr. Solly is very favourably known to the profession as a human and comparative anatomist. His present translation of Müller's work on the glands will not derogate from the good opinions which his previous productions have won for him. In the Introduction, in which Mr. Solly speaks of the work he now presents in an English garb, he observes :—

" The information which it contains, and the fundamental doctrines established by the researches which it records, cannot be too highly appreciated; and though no doubt all teachers of physiology are familiar with them, and yearly communicate them to their pupils, yet it is hardly to be expected that the great body of medical practitioners should have had the opportunity of referring to this most important volume. I hope, therefore, to present an acceptable offering to the profession, by giving an abstract of its contents. A complete translation of the whole work would not, I imagine, prove so generally useful to my professional brethren, as a faithful analysis, and I have endeavoured to add the most important observations which have appeared on this subject since its publication in 1830."

Independently of some prefatory observations, the work consists of fifteen books and an appendix. The *first* book is on the structure and development of the simple cutaneous follicles—the *second*, on the minute structure of the intestinal glands—the *third*, on the structure of peculiar excreting glands in certain animals—the *fourth*, on the structure of the glands which are appended to the organs of generation—the *fifth*, on the minute structure of the mammæ—the *sixth*, on the structure of the glands subsidiary to the organs of sense—the *seventh*, on the intimate structure of the salivary glands—the *eighth*, on the intimate structure of the pancreas—the *ninth*, on the intimate structure of the liver —the *tenth*, on the intimate structure of the kidneys—the *eleventh*, on the intimate structure of the testicle—the *twelfth*, summary of anatomical observations on the intimate structure of glands—the *thirteenth*, a natural order of glands, arranged according to their anatomical characters—the *fourteenth*, corollaries on the evolution and first formation of glands in the embryo—the *fifteenth*, physiological corollaries on secretion, particularly of glands.

The Appendix is made up of—Owen on the termination of the urinary ducts —Dr. Sprott Boyd on the mucous membrane of the stomach—Dr. Davy on the male organs of some of the cartilaginous fishes—Dr. Bischoff on the mucous membrane of the stomach—Purkinje on the same.

As the subject is too exclusively anatomical to permit us to venture on an analytical account of the work, we shall merely select those portions which will put our readers in possession of the more modern discoveries or opinions of moment to them as practitioners.

We shall take the thirteenth book which follows out the development of glands from the most simple to the most elaborate, as a key to the existing ideas on their nature.

### A NATURAL ORDER OF GLANDS, ARRANGED ACCORDING TO THEIR ANATOMICAL CHARACTERS.

#### I. GLANDS OF THE FIRST AND MOST SIMPLE ORDER.

"There are four elementary forms of the most simple gland; the cellular, the follicular, the cœcal intestinule, and blind vessels or tubuli.

1. *Crypts or cellules.*

These are pits slightly scooped out of the secreting membranes."

2. *Follicular or pedunculated vesicles.*

3. *Elongated utriculi,* or cœcal intestinules.

Here the eversion of the simple membrane is greater, but without any neck or swelling.

4. *Tubuli,* or blind vessels.

This division consists of very long prolongations, or tubuliform pouches, straight or bent, with cœcal apices. These tubes or blind vessels are sometimes twisted into clusters and convolutions, even sometimes spirally, as in the testicles of many insects. They are either simple, or united by different orifices.

1. Cryptæ Compositæ, or compound cellules; many crypts united in receptacles, or sacs.
2. Folliculi Compositi, or compound pedunculated vesicles.
3. Intestinula Cæca Composita.
4. Tubuli, or compound cœcal vessels.

#### III. GLANDS OF THE THIRD ORDER.

*Cells, Follicles, Intestinules, and compound Tubes united in a glandular bag.*

#### IV. GLANDS OF THE FOURTH ORDER.

*Glands composed of cellular tissue.*

1. Glands composed of the spongy cellular tissue, with central cells, leading to excretory ducts, but without any compound division into lobules.
2. Glands composed of spongy cellular tissue, with septa placed between the segments of the glands.
3. Glands composed of spongy cellular tissue, separated externally into lobes, with excretory ducts.
4. Glands composed of spongy cellular tissue, divided into lobes, the cells leading to the central cavities, without any proper excretory ducts.
5. A spongy web of cells in the parallel tubes of the gland.

#### V. GLANDS OF THE FIFTH ORDER.

*Lobulated Glands, composed of twig-like cæcal intestinules.*

1. A gland formed by twig-like cœcal intestinules, bound up together as fasciculi in the lobules.
2. A gland formed of ramose intestinules, arranged in a foliated manner in the lobules.
3. A gland formed by twig-like cœcal intestinules, irregularly placed.

#### VI. GLANDS OF THE SIXTH ORDER,

*Ramose branch-like excretory ducts, surrounded from beginning to end by elementary particles.*

This efflorescence is not merely terminal, but it accompanies the branches throughout, even to the most minute twigs, such as botanists call protracted efflorescence.

### VII. GLANDS OF THE SEVENTH ORDER.

*A complicated ramification in a lobulated gland, with trunks continuous and entire between the lateral branches of the excretory ducts, and the ultimate terminations of which are vesicular.*

"An entire and continuous trunk of the excretory duct, which does not terminate in ramifications, but sends off smaller branches laterally, which pursue their course continuously, through its own lateral ramification, from which lobules of different degrees of size arise. For each trunk connects the larger lobes with the lateral ramifications, and the branches form with the twigs lobules of a second order. Finally, the most minute lobules are formed out of the ultimate particles which grow upon the smallest offsets of the twigs. The origin of these lobules is explained by embryology.

To this class belong the salivary glands, the pancreas, the mammæ and lacrymal glands of many mammalia."

### VIII. GLANDS OF THE EIGHTH ORDER.

*A compound ramification in non-lobulated glands, the trunks dividing into irregular branches, with cœcal, twig-like, or vesicular terminations.*

"In this order the trunk continues through the lateral branches; it is soon lost in its own ramifications, gradually terminating in the most minute twigs. But if the whole organ be very much evolved, the whole organ puts on externally the appearance of a species of parenchyma, as in the liver of some of the higher animals, in which the liver consists of a system of small and large lobules, but offers only segments and incisions, 'Segmenta et incisiones.'"

### IX. GLANDS OF THE NINTH ORDER.

*Formed by Tubes and Vessels, and not of ramose Cœca.*

"The elements of these glands are very long tubes, of an equal diameter from their origin to their termination, either straight or winding, sometimes bifurcated from the beginning, and becoming simple after giving off their branches."

Of these M. Müller describes six forms, but we need not particularize them.

The *Twelfth Book* presents us with a very brief

### SUMMARY OF ANATOMICAL OBSERVATIONS ON THE INTIMATE STRUCTURE OF GLANDS.

The facts, says the translator, which have been brought forward demonstrate, that:—

"However much secreting organs may differ in their conformation, they exhibit a continuous series through the animal kingdom from a simple follicle, without ramifications, up to the most complicated structure; consequently that there is no distinction between the secreting organs of the invertebrata and those of the highest animals; simple tubular-shaped follicles and convoluted cœcal ducts pass by a continuous chain through the different series of animals into conglomerate glands.

Secondly, that this boundless variety in form has simply relation to an increase of secreting surface, within a circumscribed space.

Thirdly, that the sanguiferous system which conveys the materials for secretion, is nowhere continuous with the ducts which contain that secretion, and that their connexion is solely that of the *surface* of one set of vessels with the surface of the other, the secretions being eliminated through the coats of the vessels, similar to the process of secretion which is constantly going on in the air-cells of the lungs.

Fourthly, that the vascular network which surrounds the secreting ducts, whatever may be their form, is composed of blood-vessels infinitely more minute than the ducts themselves.

Fifthly, that the evolution of glands is similar in all classes of animals, and that the most complicated glands of the highest animals, originate in the embryonic stage from a single tube, like the simple secreting organs of the lowest.

Sixthly, that there is no correspondence between the form of the secreting surface and its product, as the existence of the same form in the kidneys of one animal, and the testicles of another, unequivocally demonstrates.

Seventhly, that as numerous nerves may be traced accompanying the renal arteries in the horse, and ramifying upon their surface, in the substance of the kidney, it is most probable that nerves form an important part in the structure of all glands.

The *Fifteenth Book* is made up of:—

PHYSIOLOGICAL COROLLARIES ON SECRETION, PARTICULARLY OF GLANDS.

The nature of Secretion has always puzzled physiologists, and it is left as much in the dark by Müller as he found it. But there are some phenomena on which observation throws light, and which it is right to be acquainted with.

"The only rational explanation," says Müller, "of secretion is, to consider it as a metamorphosis of the animal matter, which the blood undergoes in various ways, while circulating through the organs." The arteries terminating only in the veins the vascular circle is, pro tanto, closed, yet it appears that a proper membrane to the minutest vessels is not always to be found, and that observation teaches that new currents of blood spring up in the substance of organs, both in the embryo and the adult; in consequence of which arrangement, "the substance of any organ itself imbibes the blood, and assimilating its particles, changes them to its own peculiar substance."

"This metamorphosis, into substance, is threefold:—1. Blood is changed into the substance of different organs in *nutrition*; 2. Blood is changed into a more fluid substance, which, passing through the walls of an organ, is denominated *secretion*; 3. Blood is changed into substance, and having passed through the walls of organs, is immediately indurated."

"This last manner of secretion does not differ much from the true and genuine secretion. For these hard particles thus deposited in the walls of an organ were fluid in the first instance, and it is in this way that the hair, feathers, shells, scales, and nails are produced."

All secretion takes place on a *surface*, whether it be that of an expanded membrane, or the more complex one of a gland. No open pores are necessary or discoverable.

"It is not the blood-vessels which secrete, but the surface of the membranes in which, as in all organs, the smallest blood-vessels produce the vascular rete or rete of currents."

"These surfaces of secreting organs receive the circulating blood through the reticulated vessels, they imbibe it, are nourished by it, but they do not secrete the altered fluid particles in a liquid state from the blood-vessels, but from their own peculiar substance, which then flows out upon the membranes themselves, or in the appropriate ducts." "No one," continues our author, "among the learned, contends that the mucus in the mucous membranes is separated by the blood-vessels: truly it is the mucous membrane itself, imbued with the circulating blood, that takes up and changes the contained fluids, and melts outwardly into a diffluent mucus."

As secretion is *not* limited to the *extremities* of excretory ducts, not even a gland is necessary for secretion. Expanded membranes, glands, foliated processes, are only so many means of giving secretion effect.

